# Air Quality and Cancer Prevalence Trends across the Sub-Saharan African Regions during 2005–2020

**DOI:** 10.3390/ijerph191811342

**Published:** 2022-09-09

**Authors:** Omolola Okunromade, Jingjing Yin, Clara Ray, Atin Adhikari

**Affiliations:** 1 Department of Health Policy and Community Health, Jiann-Ping Hsu College of Public Health, Georgia Southern University, Statesboro, GA 30460, USA; 2 Department of Biostatistics, Epidemiology and Environmental Health Sciences, Jiann-Ping Hsu College of Public Health, Georgia Southern University, Statesboro, GA 30460, USA; 3 Department of Geology and Geography, College of Science and Mathematics, Georgia Southern University, Statesboro, GA 30460, USA

**Keywords:** air quality, cancer, PM_2.5_, CO_2_, air pollutants, methane, greenhouse gases, sub-Saharan region

## Abstract

Poor air quality and environmental pollution remain some of the main etiological factors leading to cancers and cancer-related deaths worldwide. As a result of human activities, deleterious airborne chemicals can be dispersed not only in the environment but also released in occupational environments and industrial areas. Air pollutants and cancer links are now established through various oxidative stress-related mechanisms and related DNA damages. Generally, ambient and indoor air pollutants have been understudied in sub-Saharan Africa (SSA) compared to other regions in the world. Our study not only highlights the deleterious effects of air pollutants in these developing countries, but it has strived to examine the trends and correlations between cancers and some air pollutants—carbon dioxide, other greenhouse gases, PM_2.5_, and human development index—in some SSA countries, where recent cancer burdens were reported as high. Our results showed strikingly higher yearly trends of cancers and above-mentioned air pollutant levels in some sub-Saharan countries during 2005–2020. Relative risks (RR) of these air pollutants-related cancer case rates were, however, below, or slightly above 1.0, or not statistically significant possibly due to other responsible and confounding factors which were not considered in our analyses due to data unavailability. We recommend new approaches to monitoring, minimizing, and creating awareness of the trends of hazardous air pollutants in sub-Saharan Africa, which will help ameliorate cancer prevalence and support the reduction in air pollution levels within regulatory limits, thereby relieving the cumulative burdens of cancers. Utilization of the findings from the study will support large-scale public health and health policy efforts on cancer management through environmental stewardship in SSA countries having the poorest outcome and the shortest survival rates from cancers.

## 1. Introduction

Globally, cancer remains a significant public health problem that needs to be addressed. The sub-Saharan region is having a faster increase in cancer incidence, the poorest outcome, and the shortest survival rates from cancer [1,2]. Most countries in the sub-Saharan regions have higher rates of cancer burden, particularly cancer types involving the prostate, breast, and cervix [2]. It has been estimated that by the year 2030 there will be a 70% increase in new cancer cases due to population growth and aging, and the annual number of deaths from cancer in Africa will be close to a million [3,4]. As of 2018, the estimated number of new cases of cancer were 752,000, which accounted for 4 percent of the global total, and 506,000 cancer deaths occurred in sub-Saharan Africa [5]. The World Health Organization (WHO) revealed that several new cancer cases have more than doubled in the African Region, from 338,000 cases reported in 2002 to almost 846,000 cases in 2020 over the past 20 years [6].

**Figure 1:**
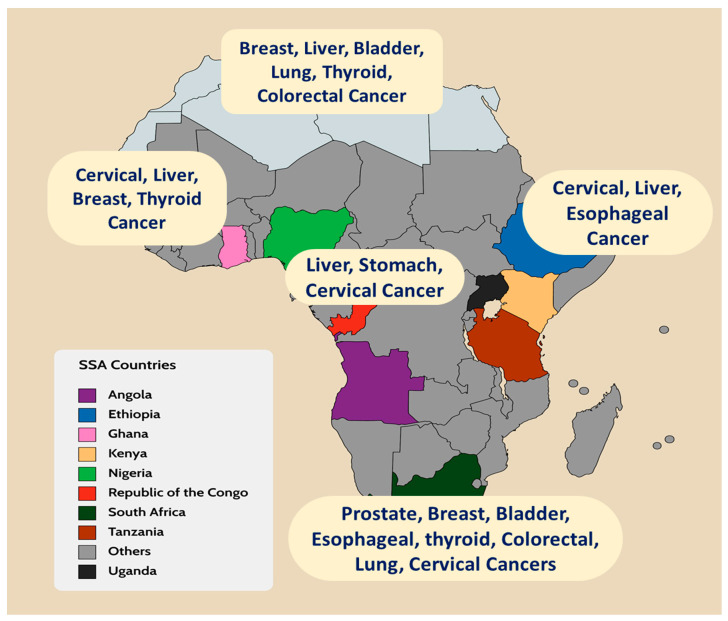
A map of Africa depicting the predominant cancer types in specific regions.

In parallel to increasing cancer cases, we have surprisingly noticed an alarming rise of air pollutants in sub-Saharan Africa [7,8,9]. These pollutants are in existence in natural or human-made forms and are present in gaseous states, liquid droplets, or solid particles. Evidence-based studies have shown that carbon monoxide (CO), sulfur dioxide (SO_2_), particulate matter (PM_2.5_, PM_10_), and nitrogen dioxide (NO_2_) specifically are responsible for this increase [10]. These pollutants are primarily from the burning of fuels [11,12]. All these pollutants could lead to increased mortality, especially lung cancer and other types of cancers such as liver cancer, colorectal cancer, bladder cancer, and kidney cancer [10]. Some cancer types were found to be predominant in some regions in SSA (Figure 1) [13].

An in-depth review below will be exploring the types of air pollution (indoor and outdoor), mechanisms of air pollution-induced cancers, the influences of air pollution on cancer and current shortcomings of air pollutants in the sub-Saharan region.

### 1.1. Indoor Air Pollution 

Exposure to household air pollutants remain a paramount issue in the field of public health [14,15]. Women and children have the highest biomass smoke exposure due to cultural practices such as indoor cooking, especially in housing with very poor air ventilation [16,17]. Some sub- Saharan regions do have homes that lack chimneys or pipes, preventing the smoke from venting outside and leading to trapped particles, which diffuse into the surroundings of homes [17,18]. Studies have shown that people inhaling trapped particles from burning of fuels during homemade cooking can be exposed up to 30,000 μg/m^3^ of PM_10_, while an average concentration throughout the day is approximately 300–5000 μg/m^3^ [11,19]. In some rural communities in Uganda, paraffin is used for lighting, and it contributes to household air pollution in rural communities [20]. Paraffin, which is one of many sources of persistent organic pollutants (POPs) in indoor environments, and POPs are toxic carbon-based chemicals transported by wind and water and have adversely affected human health and the environment around the world [12]. They accumulate in the food chain and can be generated in one country and persist for long periods in the environment. Exposures to POPs and other related compounds such as PCBs, brominated flame retardants, organochlorine pesticides, and polycyclic aromatic hydrocarbons (PAH) are regarded as a significant environmental risk factor for human cancers [12]. (EPA, 2021). Human exposure to POPs could also occur through consumption of contaminated seafood and livestock such as eggs, milk, and fish [21]. These foods are major staple diets consumed by people living in Africa, and POPs could be present in food in the raw stages, transferred from the environment, or artificially introduced during food preparation steps at home [21,22].

### 1.2. Outdoor Air Pollution 

In poor developing countries in SSA, burning biomass fuels is a more affordable and accessible alternative than liquefied petroleum gas or electricity [23]. Smoking from biomass occurs due to the combustion of different types of fuel source including burning of wood, animal dung, and crop residues undertaken to create the energy necessary for cooking and heating in many households worldwide. Biomass constitutes over 200 different compounds with significant proportions of toxic compounds such as PAH, aldehydes, free radicals, non-radical oxidizing species, and volatile organic compounds [18,23] (Gordon et al., 2014; Jain et al., 2016). Major sources of NO_2_, CO, and SO_2_ are the combustion of fossil fuel in vehicles, industry, and power generators [24]. In addition, 60% of greenhouse gas emissions come from slash-and-burn farming, in which the natural vegetation is cut down and burned to clear the land in preparation for cultivation in sub-Saharan Africa [25].

### 1.3. Mechanisms of Air Pollution-Induced Cancers

The literature echoed two main mechanisms for air pollution-induced cancers. One of these mechanisms includes DNA damage due to oxidative stress [26,27]. Reactive oxygen species are also formed in response to particulate matter and sulfur dioxide exposures, which ends up causing oxidative stress [26]. Oxidative stress eventually plays an integral role in cancer cells’ survival outcomes by interfering and promoting cell proliferation, gene mutations, and genetic instability [27]. In addition, researchers have linked the mechanism of breast, prostate, colorectal, cervical, and some other cancers to be exacerbated by NO_2_ and SO_2_ pollutants through oxidative stresses [26].

Through in vitro and cohort studies, scientists reported that inhaled air contaminated with NO_2_, and particulate matter has led to the development of proinflammatory cytokines such as interleukin-6 (IL-6) and IL-8 [27,28]. The release of these inflammatory cytokines accounts for the second mechanism for air pollution-induced inflammations leading to cancers [27]. Persistent organic pollutants, endocrine disruptors, and other indoor and outdoor pollutants interfere with hormonally responsive tissue functions through hormone signaling and cell function imbalance [29,30,31,32].

### 1.4. Exogenous and Endogenous Environmental Risks and Their Influences on Cancer Development

Several other studies noted that though it is challenging to fully understand the individualized etiological factors of cancer, the interaction of various environmental risk factors was found to have the most significant impact on cancer development [33,34].

Over the years, studies have hypothesized gaseous pollutants such as nitrogen oxides, ammonia, sulfur oxides, carbon dioxide, carbon monoxide, and ground-level ozone could be notable cancer-causing agents [35,36]. Acids, organic molecules, some metals, and coarse and fine dust are a few examples of PM, which exist in different forms and diameters categorized as coarse PM_10_ (aerodynamic diameter of ≤10 µm), ultrafine PM_0.1_ (aerodynamic diameter of ≤0.1 µm), and fine PM_2.5_ (aerodynamic diameter of ≤2.5 µm) [37]. PM_2.5_ comprises several metals and allergens such as sulfur oxides, nitrogen oxides, and organic chemicals [38]. These chemicals have the propensity of generating toxicity to cells and later lead to the induction of oxidative stress reactions as well [27].

Anderson et al. (2017) found an association between new cases of postmenopausal breast cancer and chronic exposure to ambient air pollution [39]. Findings from this study were similar to other studies which identified breast cancer cases as a result of polycyclic aromatic hydrocarbons (PAHs) originating from traffic exhaust [40,41]. Using animal models, ambient PM, and carbon black (CB) were found to play an integral role in the progression of extra-pulmonary organs’ diseases, especially in the liver, since liver microvasculature is very accessible to hepatocytes [42]. Cadmium, arsenic, and nickel may also function as carcinogenic factors for pancreatic cancer, although some other studies were yet to find any clear associations [43]. Moreover, cancer emerges as the result of disturbed cell function, which occurs when there is an accumulation of many genetic and epigenetic changes within the cell [34,44,45].

According to the World Health Organization, approximately 17% of lung cancer deaths in adults are attributable to exposure to carcinogens from air pollution caused by cooking with kerosene or solid fuels such as wood, charcoal, or coal [35]. Emissions due to the burning of living and dead vegetation have been reported to be the largest source of biomass burning emissions [25,46].

Globally, ambient PM_2.5_ air pollution was estimated to have contributed to 14.1% of all lung cancer deaths, and 265,267 lung cancer deaths in 2017 [47]. The global proportion of lung cancer deaths from ambient PM_2.5_ was second only to tobacco smoking which was reported to be 14.1% vs. 63.2% [47]. This article also emphasized that there was credible evidence of a link between outdoor ambient air pollution, and particularly PM in outdoor air, with lung cancer incidence and mortality, causing hundreds of thousands of lung cancer deaths annually worldwide [48].

### 1.5. Current Shortcomings of Cancer-Relevant Air Pollution Data from SSA Regions

**Figure 2:**
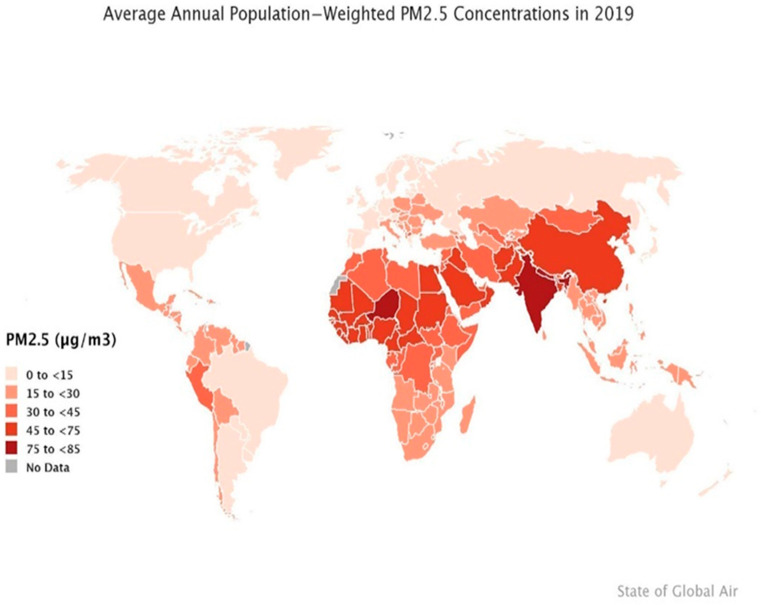
The levels of PM_2.5_ worldwide [Source: Health Effects Institute (2020)]. State of Global Air (2020). PM_2.5_ Exposure, State of Global Air (2020). Retrieved 28 November 2021, from https://www.stateofglobalair.org/air/pm [50]. (Permission was granted to use this figure on 13 July 2022 by the Health Effects Institute staff).

Given the well-documented sufficient data regarding the magnitude of air pollutants in other continents, unfortunately, there is paucity of air pollution monitoring and related cancer research in the African continent despite the rapid pace of urbanization [8]. Therefore, we set out to assess the current cancer trends in Africa and explore levels of the major primary air pollutants. Especially since most health studies on air pollutants in SSA have been majorly cross-sectional studies, which could explain the current absence of national policies in these developing countries [49]. Figure 2 below illustrates the PM_2.5_ levels worldwide and countries above WHO regulatory limits including the countries of SSA regions [50].

Our objective was to characterize, augment and bridge gaps in the state of research targeting air pollutants levels over a decade and it correlates with cancer prevalence in SSA region countries. Acknowledging the dearth of research in this field, we have prompted to answer the following research questions: (1) Does the state of air quality have any effects on cancer cases in some specific sub-Saharan countries during 2005–2020? (2) Do some pollutants have stronger association with cancer prevalence compared to others in sub-Saharan countries?

## 2. Methods

### 2.1. Data Sources

This study traced the trends of all cancer types using secondary data retrieved from the Integrated African Health Observatory and World Health Organization websites. The data source was from the Institute for Health Metrics and Evaluation [51]. IHME is a research institute working on global health statistics and impact evaluation at the University of Washington in Seattle. The IHME has a mission to evaluate the world’s most pressing health issues and to evaluate the strategies used to address them [51]. Indicators were selected as the number of cancer cases (all causes). Another data source used in this study was the secondary data set compiled from the World Bank World Development Indicators (WDI) and United Nations Development Program [52,53].

### 2.2. Country Selection

For this study, simple random sampling was used to select some SSA countries utilized and countries such as Angola, the Republic of Congo, Ethiopia, Ghana, Kenya, Nigeria, South Africa, Tanzania, and Uganda.

Data were then compiled for following SSA countries: Angola, Republic of Congo, Ethiopia, Ghana, Kenya, Nigeria, South Africa, Tanzania, and lastly Uganda with a focus on the trends of registered cancer cases. The trends in overall annual prevalence of all cancer cases in 9 countries within the years were analyzed and attempts were made to compare with major risk factors of cancer—selected air pollutants, smoking, economic development, and socioeconomic indicators such as the human development index (HDI).

### 2.3. Variables and Data Descriptions

The rationale for collecting the variables was to select indicators related to indoor and outdoor pollution over an extensive annual timespan within the years 2005 to 2020. Using panel data, pollutants such as PM_2.5_, CO_2_ (including CO_2_ emissions from liquid fuel consumption), total greenhouse gas emissions, and methane were considered. The data variables and their sources are shown below in Table 1.

### 2.4. Data Analyses Methods

Using the Statistical Analysis Software (SAS), the time series of each variable were plotted by country [54]. Descriptive statistics by year and country were computed using the Means procedure. Poisson regression models were fitted using the generalized linear model (GENMOD) procedure [55] to account for the correlation between the measurements of the variables over years within each country where country is considered as the cluster variable and autoregressive “AR(1)” was used for specifying the working correlation. The Poisson model was structured as

$$y_{i}\sim Poisson\left(u_{i}\right)\;log\left(u_{i}\right)=\beta_{0}+\eta_{1}*year_{i}+\eta_{2}*log\left(population_{i}\right)+\sum \beta_{j}x_{ij}$$

where $x_{ij}$ are jth exposure variable for the ith observation.

Modeling was performed regressing the cancer cases with log of the annual population of each country as the offset, thus we are fitting the cancer case rate, i.e., ($log\left(u_{i}/population_{i}\right))$ on the moving average of each pollutant (PM_2.5_, CO_2_, methane, and other greenhouse gases) cumulatively over the past five years and controlling for yearly trend. Then, a multiple regression model was fitted to estimate the case rate controlling for all pollutants.

## 3. Results

### 3.1. Cancer Trends Comparisons by Country, 2005–2020

**Figure 3:**
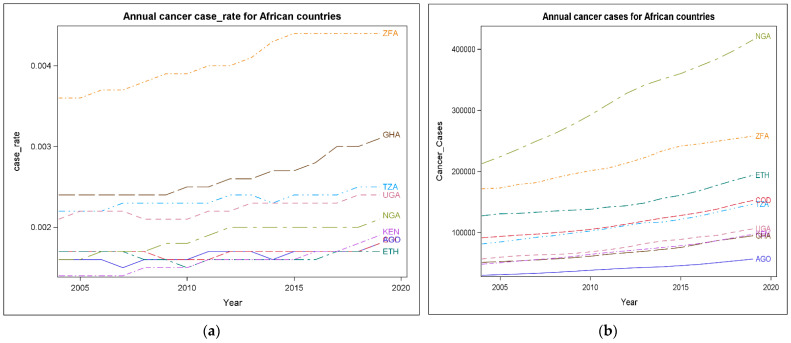
(**a**) Cancer case rate trends during 2005–2020; (**b**) cancer case trends during 2005–2020.

The trends of cancer case rates and cancer cases in the countries of Angola (AGO), Republic of Congo (COG), Ethiopia (ETH), Ghana (GHA), Kenya (KEN), Nigeria (NGA), South Africa (ZFA), Tanzania (TZA), and Uganda (UGA) were plotted in Figure 3a,b. The two line graphs showed an upward trend in most of the countries.

The annual emergence of cancer case rate in South Africa specifically was shown to have spiked higher towards the upward trends when compared to other countries (Figure 3a). Nigeria had the highest cancer cases, followed by South Africa when compared to other sub-Saharan African Countries (Figure 3b). While there was higher cancer case rate recorded for South Africa, Ghana, Tanzania, Uganda, and Nigeria, the countries Angola and Kenya showed a slight increase during 2005 to 2020 (Figure 3a).

### 3.2. Air Pollutants Levels Comparisons by SSA Countries, 2005–2020

**Figure 4:**
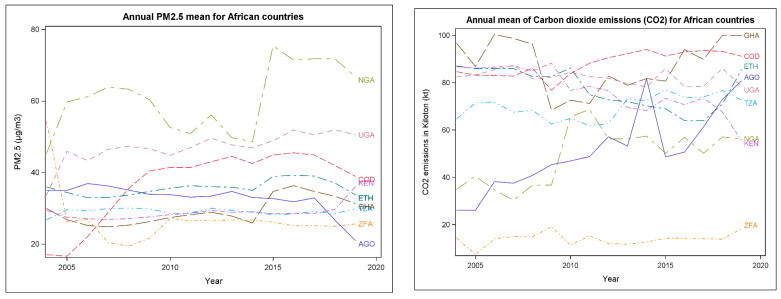

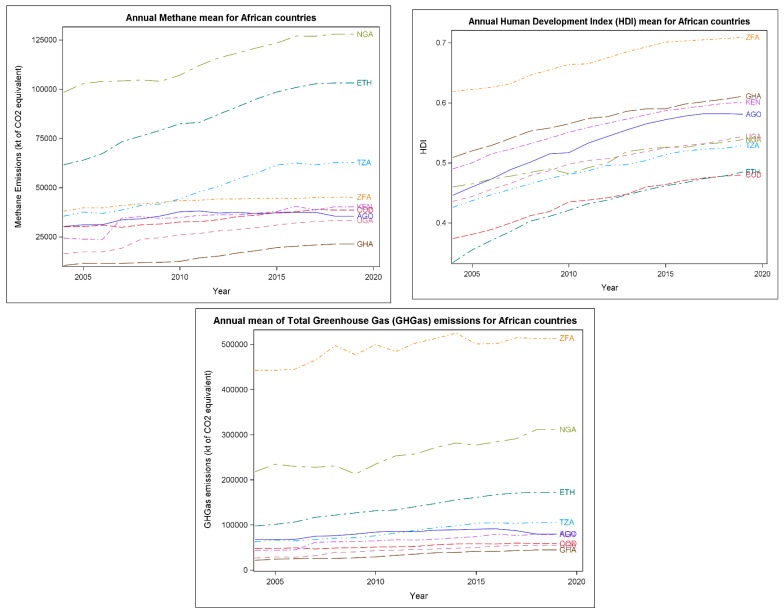
Annual pollutants levels plotted by country during 2005–2020.

**PM_2.5_**: Various variables including pollutants and risk factors—Human development Index were plotted simultaneously by countries (Figure 4). The data clearly showed that amongst all the countries that were critically examined, Nigeria constantly had the highest levels of PM_2.5_ during 2005–2020, compared to other countries. Overall, all countries were reported to have extremely high levels of PM_2.5_ in some of the years examined (Figure 4).

**CO_2_**: CO_2_ emissions in Ghana, the Republic of Congo, Tanzania, Uganda, and Angola had the highest CO_2_ emissions levels. Unlike other countries, Kenya illustrated a gradual decline in CO_2_ emission across the years, though CO_2_ levels are still relatively high. On the other hand, the level of South Africa’s CO_2_ remained low during 2005 to 2020.

**Methane, GHGAs**: The mean for other variables was also plotted below methane and GHGAs, which revealed a gradual rise annually during 2005–2020.

**HDI**: The HDI plot below revealed a transient increase during the years in all the nine countries examined. The HDI index measures essential dimensions of human development which include life expectancy measurement, access to education, and standard of living measured by Gross National Income per capita adjusted for the price level of the country [52].

Descriptive analysis results for the pollutants are presented in Table 2 and Table 3 below. The average annual concentration for PM_2.5_ from the year 2005 to 2020 ranged from 26.8 μg/m^3^ (recorded in South Africa) to 60.5 µg/m^3^ (recorded in Nigeria). Overall, mean concentrations values in all nine countries—Angola, the Republic of Congo, Ethiopia, Ghana, Kenya, Nigeria, South Africa, Tanzania, and Uganda—were exponentially high.

In addition, the average concentrations of PM_2.5_ were strikingly higher when compared to the WHO annual threshold guideline of 5 μg/m^3^ and that of the current Environmental Protection Agency’s (EPA) primary and secondary annual average standards for PM_2.5_ currently are 12.0 µg/m^3^ and 15.0 µg/m^3^, respectively [12,56].

### 3.3. The Associations between Cancer Case Rates and Air Pollutants Levels

We included year as a covariate in all models to adjust for the trend effect that we observed in the descriptive time series plots. Case rates were found to be significantly changing over the year with *p*-values < 0.05 in all of the models regressing on each pollutant. The results of associations between each pollutant and cancer case rate are listed in Table 4. The analyses showed RR estimates along with its 95% confidence limits by single-pollutant model (Table 4) and multi-pollutant multivariable linear regression models adjusted for all pollutants such as PM_2.5_, CO_2_, methane, and GHGA_s_, including HDI (Table 5).

Associations for all pollutants were found not statistically significant in the single pollutant models because *p*-values were greater than the significance level of 0.05 (Table 4).

In multi-pollutant regression models, each of the pollutant variables was additionally adjusted with all other pollutants and year (Table 5). Association for PM_2.5_, CO_2_, HDI and greenhouse gases were not statistically significant because their *p*-values were greater than the significance level of 0.05 (Table 5). Methane exhibited *p*-values = 0.0427, slightly less than 0.05 thus statistically significant, but RR was <1. CO_2_ showed positive but not significant association with cancer case rate, both in single- and multi-pollutant models (Table 4).

## 4. Discussion

To our knowledge, this study has substantially contributed to the current knowledge gaps and the limited literature on cancers and air quality relationship in the countries of the SSA region. Our study analyzed air pollutants trends and cancer trends in SSA countries and associations between air pollutant levels and cancer prevalence in those countries. Despite the WHO air quality recommendations that PM_2.5_ levels annual mean should not exceed 5 μg/m^3^ and 24 h mean should not exceed 15 μg/m^3^, our study revealed that all countries examined exceeded these recommendations regarding PM_2.5_ levels [56]. We found the levels of greenhouse gas emissions containing carbon dioxide, nitric oxide, and fluorinated gases (F-gases) are gradually declining in South Africa, Tanzania, Angola, the Republic of Congo, Nigeria, and Uganda. However, they still remain higher than WHO recommended standards [56].

Our work found that South Africa is heavily influenced by the air pollutants from domestic and industrial sectors. The findings from this study showed that South Africa is the largest emitter of CO_2_ in sub-Saharan Africa to date. In addition, we found that South Africa and Nigeria have markedly produced high levels of emissions consistently over the last decade. In addition, this study revealed statistically significant associations between methane levels with cancer cases but RR values were <1, which is consistent with the findings from a previous study that did not find significant association between methane and bowel cancers in the African population [57]. HDI on the other hand was reported to be primarily associated with prostrate and urologic cancers [58] and our RR value for HDI was <1 because we have considered all cancers together.

In addition to the markedly rising trend in the poor air quality across the years in some SSA countries—Nigeria, Tanzania, South Africa, and Ghana—our results have also simultaneously shown how cancer prevalence has spiraled in these countries.

### 4.1. Public Health Implications

In traditional rural settings in countries such as Uganda, Kenya, Nigeria, Tanzania, South Africa, and many other countries in SSA, women and children collect firewood for heating, cooking, and as a source of lighting like kerosene lamps [16,59]. CO_2_ and other chemicals generated from these household activities and cooking needs are directly causing exposure to women, echoing the voices of women as one of the vulnerable groups to air pollutants [59]. Perhaps, these existing social norms and cultural behaviors explain the increases in breast cancer cases in the SSA regions [60].

Countries of SSA regions will continually face environmental challenges due to the ongoing fast urbanization with transformation if no actions are taken [8,61]. Subsequently, air quality in SSA cities has deteriorated owing to rapid population growth, and industrial expansion in these areas from natural and manmade pollutants in the form of solid, liquid droplets, or gases that contaminate air quality. The quality of air has long been recognized as one of the major threats to cancer and overall human public health [61]. Air pollution has been classified as carcinogenic to humans [62]. The International Agency for Research on Cancer (IARC) also emphasized that outdoor air pollution and particulate matter (PM) in outdoor air pollution is carcinogenic to humans [62,63,64,65]. Major sources of chemicals and emissions such as domestic burning, household cooking, power generation, vehicle transportation, chemicals released from industries, and many more, have led to the deteriorated air quality in sub-Saharan countries considerably exceeding the WHO’s recommended safety air-quality guidelines [56]. In 2020, the IARC in WHO reported 56,802 and 78,899 cancer-related deaths in South Africa and Nigeria, respectively, with increasing trends in lung and bladder cancers and other chronic diseases [65].

### 4.2. Limitations

This study encountered several limitations. Firstly, the cancer data available may have been under-reported. Due to individual and interpersonal challenges such as fear and stigmatization amongst people in these SSA regions, there could have been cancer-related deaths at traditional families that were missed and never reported. At the organizational level, limited/lack of functional standardized cancer registries in many health care facilities in these SSA countries has also increased the problems with underreporting of many cancer-related cases and deaths. Thus, due to data underreportedness, it is reasonable to consider that there are higher than estimated cancer-related prevalence and deaths over time. Secondly, due to data source constraints, data for other chemicals such as radon, benzene, PM_10_, and asbestos, which are known to have links with leukemia, liver, and colon cancers from previous studies, were not analyzed in our study. Thirdly, we attempted to consider cigarette smoking as a potential confounding factor to avoid spurious associations. However, due to insufficient data availability, we were not able to consider this. On the other hand, efforts were made to control for confounding variables with the limited data in our multiple regression analyses. Another limitation is lack of detailed information on environmental monitoring methods. The environmental data used in this article were collected from various international databases. Unfortunately, specific information on individual tools and methods of measurements were not described in the databases.

### 4.3. Future Recommendations

For future follow-ups, it is recommended that specific culturally sensitive intervention campaigns need to be implemented for the vulnerable populations who are long-term exposed to hazardous airborne chemicals and endocrine disruptors over last several decades. Policymakers in sub-Saharan countries need to create and implement realistic and appropriate strategies targeting the reduction in these environmental risks to children, mothers, roadside hawkers, professional motorcyclists, bus drivers, and other vulnerable populations most prone to daily chemicals and air pollutants. A study conducted in Brazil demonstrated that taxi driver’s oxidative and inflammatory status were linked to occupational exposure to urban air pollution and contributed to disruptive cellular responses, leading to increased cancer development and progression in this cohort [66]. Our study recommends that research promotion and research capacity building on air pollution should be reinforced in research institutions in the SSA regions. In addition, the governmental sector in these SSA regions need to assign unceasing long-term funding for managing air pollutants and improving air quality.

Substantial awareness programs and discussions concerning the need for detailed databases containing high-quality reported cancer data for the sub-Saharan region is also needed to guide interventions aimed at improving responsiveness on cancer incidence, prevalence, and mortality and monitoring actual cancer outcomes in sub-Saharan Africa.

## 5. Conclusions

Our findings conclude that high levels of PM_2.5_ in all the sub-Saharan countries above the regulatory limits persisting during 2005–2020. This finding is important because PM_2.5_ is a well-known cause of epigenetic and microenvironmental alterations in lung cancer [67]. We have also found increasing trends of carbon dioxide, PM_2.5_, methane, and other greenhouse gases as well as cancer prevalence on most occasions in our selected SSA countries (Angola, Republic of Congo, Ethiopia, Ghana, Kenya, Nigeria, South Africa, Tanzania, and Uganda). RR values of these air pollutants-related cancer case rates were, however, below, or slightly above 1.0, or not statistically significant possibly due to other responsible and confounding factors which were not considered in our analyses due to data unavailability. We recommend further public health and health policy interventions for controlling air pollutants and cancers in SSA countries. Africa must invest in sustainable cancer prevention programs in SSA regions, and realistic measures should be considered to curtail and lessen PM_2.5_ emissions urgently.

## Figures and Tables

**Table 1:** Description of variables.

Variable	Source
Particulate matter PM_2.5_ (Mean annual Exposure in micrograms per cubic meter)	WBDI *
Total greenhouse gas emissions Kiloton of CO_2_ equivalent (GHGas)	WBDI *
Methane Emissions kt of CO_2_ equivalent (CH_4_)	WBDI *
Carbon dioxide emissions kiloton (CO_2_)	WBDI *
Cancer Prevalence (cancer cases and case rate)	IHME
Human Development Index (HDI)	UNDP

WBDI: World Bank Development Indicator; IHME: Institute for Health Metrics and Evaluation; UNDP: United Nations Development Programme. * Cancer prevalence (all cases) is defined as the number of people affected with any kind of cancer each year.

**Table 2:** Descriptive statistics by year 2005–2020. The MEANS Procedure across nine countries.

Year	No Obs	Variables	Mean	Median	Variance		Variables	Mean	Median	Variance
2004	9	Cancer Cases	96,885.33	81,490.00	3,836,512,968	2005	Cancer Cases	100,077.67	84,698.00	4,117,779,881
		Population	49,976,022.89	37,379,766.00	1.32		Population	51,320,652.78	38,450,323.00	1.39
		Case rate	0.002	0.001	4.52		Case rate	0.00	0.0017000	4.55
		PM_2.5_	34.14	33.60	115.39		PM_2.5_	33.53	29.60	159.60
		CO_2_	64.16	82.460	955.24		CO_2_	63.39	83.18	935.13
		Methane	38,355.56	30,260.00	717,212,178		Methane	39,785.56	31,110.00	784,609,928
		HDI	0.45	0.44	0.01		HDI	0.46	0.46	0.006
		GHGas	114,265.56	62,620.00	18,689,196,428		GHGas	117,437.78	66,440.00	18,978,634,344
2006	9	Cancer Cases	103,584.56	88,435.00	4,513,505,625	2007	Cancer Cases	106,937.44	92,282.00	4,934,781,160
		Population	52,710,351.33	39,548,666.00	1.47		Population	54,232,659.33	40,681,416.00	1.54
		Case rate	0.002	0.001	4.85		Case rate	0.002	0.002	5.025
		PM_2.5_	33.87	29.26	147.25		PM_2.5_	34.49	29.93	176.78
		CO_2_	66.65	82.97	894.18		CO_2_	65.77	82.77	917.32
		Methane	40,260.00	31,180.00	822,985,975		Methane	42,856.67	34,590.00	820,484,675
		HDI	0.47	0.47	0.01		HDI	0.49	0.48	0.006
		GHGas	117,968.89	64,640.00	19,003,282,311		GHGas	124,262.22	67,750.00	20,148,814,194
2008	9	Cancer Cases	110,586.44	95,123.00	5,432,909,761	2009	Cancer Cases	114,718.00	99,147.00	6,009,519,804
		Population	55,725,810.44	41,853,944.00	1.62		Population	57,172,932.11	43,073,830.00	1.71
		Case rate	0.002	0.002	5.24		Case rate	0.002	0.0018000	5.71
		PM_2.5_	35.20	33.69	172.05		PM_2.5_	35.71	33.91	143.15
		CO_2_	65.78	81.64	789.63		CO_2_	62.41	68.36	566.21
		Methane	44,414.44	35,280.00	812,477,578		Methane	45,040.00	35,590.00	817,753,375
		HDI	0.50	0.48	0.006		HDI	0.51	0.49	0.006
		GHGas	130,320.00	70,490.00	22,750,733,400		GHGas	127,467.78	71,630.00	20,347,260,019
2010	9	Cancer Cases	119,184.00	102,703.00	6,615,432,444	2011	Cancer Cases	124,259.67	106,875.00	7,318,883,180
		Population	58,762,802.89	44,346,532.00	1.81		Population	60,404,539.22	45,673,520.00	1.91
		Case rate	0.002	0.002	5.94		Case rate	0.002	0.002	5.93
		PM_2.5_	35.50	33.80	81.71		PM_2.5_	35.63	33.10	79.83
		CO_2_	65.82	72.62	572.02		CO_2_	65.51	71.18	490.66
		Methane	46,748.89	37,730.00	868,274,361		Methane	48,243.33	38,100.00	926,473,350
		HDI	0.51	0.50	0.01		HDI	0.52	0.50	0.01
		GHGas	134,881.11	75,950.00	22,597,295,686		GHGas	136,954.4	81,790.00	21,454,099,328
2012	9	Cancer Cases	129,608.78	111,872.00	8,137,820,528	2013	Cancer Cases	134,992.44	116,079.00	8,804,454,974
		Population	62,096,530.44	47,053,033.00	2.01		Population	63,835,400.56	48,483,132.00	2.12
		Case rate	0.002	0.002	5.88		Case rate	0.002	0.002	6.344
		PM_2.5_	37.00	33.40	106.76		PM_2.5_	36.14	34.70	80.76
		CO_2_	65.83	72.73	548.80		CO_2_	65.16	71.95	542.45
		Methane	49,800.00	37,110.00	1,008,354,375		Methane	51,391.11	37,420.00	1,063,888,136
		HDI	0.53	0.51	0.005		HDI	0.54	0.52	0.01
		GHGas	141,530.00	84,930.00	23,045,993,400		GHGas	147,126.67	88,410.00	24,022,736,550
2014	9	Cancer Cases	140,103.00	117,310.00	9,396,953,826	2015	Cancer Cases	144,637.67	121,669.00	9,861,388,940
		Population	65,616,581.44	49,960,563.00	2.24		Population	67,435,979.44	51,482,638.00	2.36
		Case rate	0.002	0.002	7.55		Case rate	0.0022667	0.002	7.95
		PM_2.5_	35.167	33.00	76.44		PM_2.5_	39.81	34.70	237.91
		CO_2_	68.49	72.42	544.14		CO_2_	65.57	73.32	582.68
		Methane	52,857.78	37,000.00	1,133,143,869		Methane	54,531.11	37,900.00	1,183,168,511
		HDI	0.54	0.52	0.005		HDI	0.55	0.53	0.01
		GHGas	151,693.33	88,980.00	25,232,211,650		GHGas	150,818.89	90,620.00	22,649,329,561
2016	9	Cancer Cases	149,847.78	126,784.00	10,303,690,961	2017	Cancer Cases	155,801.56	133,507.00	10,774,916,296
		Population	69,292,760.89	53,049,231.00	2.49		Population	71,185,333.44	54,660,345.00	2.62
		Case rate	0.002	0.002	7.91		Case rate	0.002	0.002	8.04
		PM_2.5_	39.80	36.30	215.70		PM_2.5_	39.62	34.70	211.81
		CO_2_	66.12	70.66	592.80		CO_2_	155,801.56	73.53	571.50
		Methane	55,851.11	40,480.00	1,247,608,986		Methane	71,185,333.44	38,830.00	1,257,685,936
		HDI	0.55	0.53	0.01		HDI	0.002	0.53	0.01
		GHGas	153,366.67	91,240.00	22,761,038,800		GHGas	39.62	87,180.00	24,168,822,528
2018	9	Cancer Cases	162,460.56	140,179.00	11,384,224,521	2019	Cancer Cases	169,066.89	146,541.00	12,137,845,441
		Population	73,107,960.89	56,313,444.00	2.76		Population	75,053,704.11	58,005,461.00	2.90
		Case rate	0.002	0.002	7.93		Case rate	0.002	0.002	7.56
		PM_2.5_	38.44	33.34	228.78		PM_2.5_	37.07	33.71	194.24
		CO_2_	70.92	72.93	633.89		CO_2_	70.91	78.26	605.45
		Methane	56,421.11	40,250.00	1,279,658,811		Methane	56,421.11	40,250.00	1,279,658,811
		HDI	0.56	0.54	0.01		HDI	0.56	0.54	0.005
		GHGas	157,681.11	79,730.00	24,828,444,611		GHGas	157,681.11	79,730.00	24,828,444,611

Abbreviations: No Obs, number of observations in nine sub-Saharan countries; HDI, Human Developmental Index; PM_2.5_, particles with aerodynamic diameter ≤ 2.5 μm; CO_2_ emissions, carbon dioxide emissions stemming from the burning of fossil fuels, cement manufacturing. consumption of solid, liquid, and gas fuels and gas flaring; Methane, emissions produced while transporting coal, natural gas, and oil; GHGas, total greenhouse gas emissions; All pollutants were observed from January to December.

**Table 3:** Descriptive statistics by country using the MEANS Procedure over 16 years.

Country	N Obs	Variables	Mean	Median	Variance	Country	Mean	Median	Variance
AGO	16	Cancer Cases	41,366.13	40,983.00	66,758,248.12	COG	115,626.88	111,594.00	379,718,492
		Population	24,992,933.81	24,664,292.50	1.68		68,656,459.50	67,887,950.00	1.16
		Case rate	0.002	0.0016500	5.33		0.002	0.002	2.50
		PM_2.5_	32.82	33.60	15.27		36.84	41.40	100.32
		CO_2_	50.99	48.66	280.77		87.95	89.36	26.44
		Methane	35,377.50	36,145.00	6,680,993.33		34,074.38	33,200.00	11,458,212.92
		HDI	0.53	0.54	0.002		0.44	0.44	0.001
		GHGas	80,915.63	81,925.00	66,932,506.25		53,124.38	51,640.00	22,428,746.25
ETH	16	Cancer Cases	150,637.56	142,945.50	438,435,539	GHA	68,339.13	65,899.50	199,393,520
		Population	92,125,083.19	91,433,455.00	1.46		25,737,389.75	25,692,083.50	8.48
		Case rate	0.002	0.002	3.83		0.003	0.003	5.90
		PM_2.5_	35.71	35.75	4.12		29.18	28.00	14.36
		CO_2_	77.45	78.71	72.75		87.41	88.30	122.80
		Methane	85,531.88	85,160.00	207,259,283		15,581.88	14,765.00	16,768,442.92
		HDI	0.43	0.44	0.002		0.57	0.58	0.001
		GHGas	138,905.00	136,870.00	671,075,560		33,592.50	33,870.00	66,632,273.33
KEN	16	Cancer Cases	69,693.88	68,938.00	223,010,639	NGA	313,308.06	318,930.50	4,217,375,062
		Population	43,887,246.50	43,760,869.50	2.94		166,198,459	165,016,942	4.38
		Case rate	0.002	0.002	2.20		0.002	0.002	2.76
		PM_2.5_	28.82	28.60	4.53		60.55	60.80	88.04
		CO_2_	76.85	76.66	87.45		49.25	53.16	143.04
		Methane	34,600.63	36,030.00	31,905,686.25		114,047.50	113,905.00	112,469,660
		HDI	0.56	0.56	0.001		0.5013750	0.50	0.00
		GHGas	65,332.50	66,755.00	146,965,153		258,021.25	255,325.00	1,055,559,958
TZA	16	Cancer Cases	110,293.38	109,373.50	396,206,282	UGA	77,633.31	74,081.50	250,870,919
		Population	46,875,990.25	46,363,276.50	4.30		34,594,752.63	34,017,736.00	3.02
		Case rate	0.002	0.002	9.1679		0.002	0.002	1.03
		PM_2.5_	29.07	29.18	0.76		47.06	47.16	18.88
		CO_2_	69.69	71.62	26.14		82.23	82.31	9.33
		Methane	49,790.00	49,240.00	111,200,027		26,272.50	27,360.00	35,392,206.67
		HDI	0.49	0.49	0.001		0.50	0.51	0.001
		GHGas	85,213.75	84,870.00	279,615,798		43,065.00	44,685.00	95,303,026.67
ZFA	16	Cancer Cases	213,399.56	209,662.50	925,988,671				
		Population	52,642,323.13	52,418,209.00	1.35				
		Case rate	0.004	0.004	9.58				
		PM_2.5_	26.84	26.20	59.48				
		CO_2_	13.88	14.13	7.27				
		Methane	42,931.88	43,900.00	5,078,056.25				
		HDI	0.67	0.67	0.001				
		GHGas	490,035.00	500,405.00	750,589,787				

Abbreviations: N Obs, number of observation in nine sub-Saharan countries; HDI, Human Developmental Index; PM_2.5_, particles with aerodynamic diameter ≤ 2.5 μm; CO_2_ emissions, carbon dioxide emissions. All pollutants were observed from January to December. Angola (AGO), Republic of Congo (COG), Ethiopia (ETH), Ghana (GHA), Kenya (KEN), Nigeria (NGA), South Africa (ZFA), Tanzania (TZA), and Uganda (UGA).

**Table 4:** Regression model estimates for each pollutant controlling for yearly trend.

Parameter	RR Estimate	95% Confidence Limits of RR		Z	Pr > |Z|
PM_2.5_	0.9997	0.9986	1.0009	−0.50	0.6190
CO_2_	1.0006	0.999	1.0023	0.73	0.4644
Methane	0.9979	0.9953	1.0005	−1.55	0.1207
HDI	0.5158	0.0349	7.6162	−0.48	0.6298
GH	0.9997	0.9979	1.0016	−0.27	0.7879

Abbreviations: HDI, Human Developmental Index; PM_2.5_, particles with aerodynamic diameter ≤ 2.5 μm; CO_2_ emissions, carbon dioxide emissions; RR, relative risk. Significance level used: 0.05. All pollutants were observed from January to December.

**Table 5:** Multiple regression results considering all selected air pollutants controlling for yearly trend.

Variable	RR Estimate	95% Confidence Limits of RR		Z	Pr > |Z|
Year	1.0290	1.0044	1.0541	2.31	0.0206
PM_2.5_	0.9999	0.9981	1.0018	−0.07	0.9408
CO_2_	1.0000	0.9984	1.0016	−0.01	0.9906
Methane	0.9969	0.9939	0.9999	−2.03	0.0427
HDI	0.3160	0.0435	2.2980	−1.14	0.2551
GH	1.0005	0.9990	1.0020	0.61	0.5437

Abbreviations: HDI, Human Developmental Index; PM_2.5_, particles with aerodynamic diameter ≤ 2.5 μm; CO_2_ emissions, carbon dioxide emissions; RR, relative risk. Significance used 0.05. All pollutants were observed from January to December.

## Data Availability

Data collected in this study are all contained within the article.
